# Metabolomic analyses for atherosclerosis, diabetes, and obesity

**DOI:** 10.1186/2050-7771-1-17

**Published:** 2013-04-01

**Authors:** Fuyong Du, Anthony Virtue, Hong Wang, Xiao-Feng Yang

**Affiliations:** 1 Department of Pharmacology, Temple University School of Medicine, Philadelphia, PA 19140, USA; 2 Cardiovascular Research Center and Department of Pharmacology, Temple University School of Medicine, 3500 North Broad Street, MERB 1059, Philadelphia, PA 19140, USA; 3 Thrombosis Research Center, Temple University School of Medicine, Philadelphia, PA 19140, USA

**Keywords:** Metabolomics analysis, Atherosclerosis, Diabetes, Obesity, Metabolites

## Abstract

Insulin resistance associated with type 2 diabetes mellitus (T2DM), obesity, and atherosclerosis is a global health problem. A portfolio of abnormalities of metabolic and vascular homeostasis accompanies T2DM and obesity, which are believed to conspire to lead to accelerated atherosclerosis and premature death. The complexity of metabolic changes in the diseases presents challenges for a full understanding of the molecular pathways contributing to the development of these diseases. The recent advent of new technologies in this area termed “Metabolomics” may aid in comprehensive metabolic analysis of these diseases. Therefore, metabolomics has been extensively applied to the metabolites of T2DM, obesity, and atherosclerosis not only for the assessment of disease development and prognosis, but also for the biomarker discovery of disease diagnosis. Herein, we summarize the recent applications of metabolomics technology and the generated datasets in the metabolic profiling of these diseases, in particular, the applications of these technologies to these diseases at the cellular, animal models, and human disease levels. In addition, we also extensively discuss the mechanisms linking the metabolic profiling in insulin resistance, T2DM, obesity, and atherosclerosis, with a particular emphasis on potential roles of increased production of reactive oxygen species (ROS) and mitochondria dysfunctions.

## Introduction

**Figure 1:**
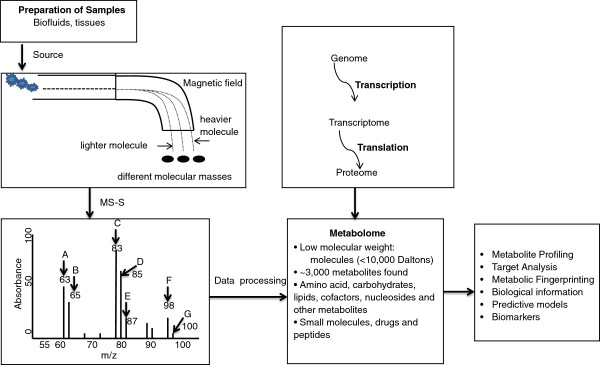
**The work flow of Mass Spectrometry Schematic (MS-S) for metabolome analysis.** The left panel depicts a triple-collector system arranged to analyze molecules and an example of total mass spectrum for the compounds A, B, C, D, E, F, and G in a reaction. The right panel outlines the relationship between the genome, proteome, and metabolome, as well as the significances of metabolomics.

The metabolome is the terminal product downstream from the genome, transcriptome, and proteome, and consists of the total complement of all the low-molecular-weight molecules (metabolites) in a cell, tissue, or organism [1,2]. Metabolomics is well defined as a technology aiming to measure/profile metabolite changes present within a cell, tissue, or organism in response to a genetic alteration or pathophysiological stimuli [3,4]. Downstream of transcriptional, posttranscriptional, translational, and posttranslational processes (Figure 1), metabolites serve as the most proximal reporters of alteration in the body/tissue/cells in response to a disease process. Measurement of time-related metabolic changes in animal models in response to genetic manipulation can be used to define the varying phenotypes observed [5]. In general, metabolomics methodologies fall into two distinct groups: untargeted metabolomics, an intended comprehensive analysis of all the measurable identities in a sample including chemical unknowns; and targeted metabolomics, the measurement of defined groups of chemically characterized and biochemically annotated metabolites. The results of targeted metabolomics studies have demonstrated both feasibility and flexibility across physiological, pathological, interventional, and epidemiological human studies. In addition to the identifications of metabolites in the process of disorders, metabolomics also has the groups of well-established web-based databases available. These databases have linked the identified metabolites or compounds with specific disorders and physiological characteristics (Tables 1, 2) and they also have well defined the biological pathways or genome, transcriptome and proteome underlining the change of metabolites (Tables 3, 4). As a result, the new methodologies of metabolomics have provided invaluable tools for the examination of these interactions on a global scale, and offer an alternative means of investigation to that of some of the more reductionist molecular biology approaches.

**Table 1:** List of compound and disease/physiology databases

**Link of the databases**	**Teams or specificity**	**Numbers of metabolites**	**Data included**
ChEBI ( http://www.ebi.ac.UK/chebi/)	Chemical Entities of Biological Interest; Small chemical compounds	More than 15,500 chemical entities	Structure and nomenclature information
ChemSpider ( http://www.chemspider.com/)	An aggregated database of organic molecules	More than 20 million compounds	Search utilities and a large number of calculated physicochemical property values.
KEGG Glycan ( http://www.genome.jp/kegg/glycan/)	A collection of experimentally determined glycan structures	More than 11,000 glycan structures from a large number of eukaryotic and prokaryotic	Glycan structures
METAGENE ( http://www.metagene.de/)	A knowledgebase for inborn errors of metabolism	431 genetic diseases	Information about the disease, genetic cause, treatment and the characteristic metabolite concentrations or clinical test used for the diagnosis of disease
OMIM ( http://www.ncbi.nlm.nih.gov/omim/)	Online Mendelian Inheritance in Man: a comprehensive compendium of human genes and genetic phenotypes	Contain information on all known Mendelian disorders and over 12,000 genes	The relationship between phenotype and genotype
OMMBID ( http://www.ommbid.com/)	An web-accessible book/encyclopedia describing the genetics, metabolism, diagnosis and treatment of metabolic disorders	Hundreds of metabolic disorders	Extensive reviews, detailed pathways, chemical structures, physiological data and tables
PubChem ( http://pubchem.ncbi.nlm.nih.gov/)	Structures and activities of small organic molecules	More than 19 millions	Structure, nomenclature physic-chemical data

**Table 2:** List of drug metabolic databases

**Link of the databases**	**Teams or specificity**	**Numbers of metabolites**	**Data included**
DrugBank ( http://www.drugbank.ca/)	A blended bioinformatics and cheminformatics database of drugs and their targets	~4,800 drug entries (>1,350 FDA*^1^-approved small molecule drugs, 123 biotech drugs, 71 nutraceuticals and >3,243 experimental drugs.	Chemical, pharmacological and pharmaceutical data; sequence, structure, and pathway information
PharrmGKB ( http://www.pharmgkb.org/)	A central repository for genetic, genomic, molecular and cellular phenotype data and clinical information of people who have participated in pharmacogenomics research study	>20,000 genes;	Data of clinical and basic pharmacokinetic and pharmacogenomic research in the cardiovascular, pulmonary, cancer, pathways, metabolic and transporter domains
		>3,000 diseases;	
		>2,500 drugs;	
		~ 53 pathways	
		~470 genetic variants affecting drug metabolism	
STITCH ( http://stitch.embl.de/)	Search tool for interactions of chemicals	More than 68,000 different chemicals including 2,200 drugs, and connects them to 1.5 million genes across 373 genomes	Information about interaction of metabolic pathways, crystal structures, binding experiments and drug-target relationships
SuperTarget ( http://insilico.charite.de/supertarget)	A core dataset of drug-target relations	~7,300 relations to 1,500 drugs	Drug-target relations; tools for 2D*^2^ drug screening and sequence comparison of the targets
Therapeutic Target DB ( http://xin.cz3.nus.edu.sg/group/ttd/ttd.asp)	A therapeutic target database	1,535 targets and 2,107 drugs/ligands	Information about the known therapeutic protein and nucleic acid targets; the targeted disease conditions; the pathway information; and drug/ligands of targets

Abbreviations: *1FDA: US food and drug administration; *2-D: 2 dimensional.

**Table 3:** List of metabolic pathway databases

**Link of the databases**	**Teams or specificity**	**Numbers of metabolites**	**Data included**
BioCyc ( http://humancyc.org/)	Pathway/Genome database	371 pathways	Tier 1: metabolites, enzymes activators/inhibitors and cofactors; Tier 2 and Tier 3: computationally predicted pathways and gene codes.
HumanCyc ( http://biocyc.org/)	Bioinformatics database	28,783 genes	Metabolic pathways and the human genome
KEGG ( http://www.genome.jp/kegg/)	Kyoto Encyclopedia of Genes and Genomes	372 reference pathways, >15,000 compounds, 7,742 drugs and ~11,000 glycan structures	Metabolic pathways hyperlinked to metabolite and protein/enzyme information
Metacyc ( http://metacyc.org/)	Data base of nonredundant, experimentally elucidated metabolic pathways	More than 1,100 pathways of more than 1,500 different organisms	Pathways of both primary and secondary metabolisms as well as associated compounds, enzymes, and genes
Reactome ( http://www.reactome.org/)	A curated, peer-reviewed knowledge base of biological pathways	More than 2,700 proteins, 2,800 reactions and 860 pathways for humans	Metabolic pathways, protein trafficking and signal pathways

**Table 4:** List of comprehensive metabolomic databases

**Link of the databases**	**Teams or specificity**	**Numbers of metabolites**	**Data included**
BIGG ( http://bigg.ucsd.edu)	A metabolic reconstruction of human metabolism	1,496 ORFs*^1^, 2,004 proteins, 2,766 metabolites, and 3,331 metabolic and transport reactions	Literature-based genome-scale metabolic reconstruction
BinBase ( http://bigg.ucsd.edu/)	A GC-TOF*^2^ metabolomic database	Unknown	Metabolomic database
HMDB ( http://www.hmdb.ca)	Human Metabolome Database;	More than 6,500;	Chemical data; Clinical data; Molecular Biology/Biochemistry data
	Small molecule metabolites	Link to ~ 1,500 protein and DNA sequences	
SetupX ( http://bigg.ucsd.edu/; http://fiehnlab.ucdavis.edu:8080/m1/readme.jsp)	A web-based metabolomics LIMS*^3^	unknown	Display of GC-MS*^4^ metabolomic data
SMPDB ( http://www.smpdb.ca/)	The small molecule pathway database	More than 350 small molecule pathways	Human metabolic pathways, metabolic disease pathways, metabolite signaling pathways and drug-action pathways
SYSTOMONAS ( http://fiehnlab.ucdavis.edu:8080/m1/login.jsp)	Systems biology of pseudomonas--systems biology studies of Pseudomonas species.	Extensive	Transcriptomics, proteomic, metabolomic and metabolic reconstructions

Abbreviations: *1ORFs: open reading frames; *GC-TOF: gas chromatography/time-of-flight; *3 LIMS: laboratory information management system; *4 GC/MS: Gas chromatograph/ mass spectrometry.

Analytical approaches applied to identify changes in the concentrations and fluxes of endogenous metabolites include, but are not limited to, ^1^H and ^13^C nuclear magnetic resonance (^1^H- and ^13^C-NMR) spectroscopy, gas chromatography-mass spectrometry (GC-MS), gas chromatography-flame ionization detection (GC-FID), direct infusion-mass spectrometry (DI-MS), and liquid chromatography-mass spectrometry (LC-MS). Figure 1 shows how metabolomic technology (MS) works and separates the molecules based on their different masses in a typical reaction (Figure 1). As an addition to other metabolomic databases listed in the tables, the spectral database includes almost all spectrometries applied in the metabolomics research (Table 5). However, due to the wide range of metabolites and the dynamic nature of their concentrations within the cell, a complete analysis of the metabolome has thus far eluded description even when a range of analytical approaches have been employed [6]. In addition, metabolomics has been increasingly applied to the biomarker discovery of cardiovascular diseases, diabetes, and early changes of metabolites in obesity through metabolic profiling in patients with metabolic syndrome [7-9], MS-based metabolic profiling of acylcarnitines species in insulin resistance animal models [10], cellular lipid signaling [11], as well as profiling amino acids and acylcarnitines in obesity individuals [12]. Although various methodologies have been employed, two technologies have prevailed as the core methodologies of metabolite profiling: NMR [13] and MS, with the latter coupled to an array of separation techniques including GC and LC.

**Table 5:** List of spectral databases

**Link of the databases**	**Teams or specificity**	**Numbers of metabolites**	**Data included**
BML-NMR ( http://www.bml-nmr.org)	The Birmingham Metabolite Library Nuclear Magnetic Resonance (NMR) database	3,328 NMR*^1^ spectra of 208 common metabolite standards.	2-D ^1^HJ*^2^ -resolved spectra and 1-D ^1^H spectra
BMRB ( http://www.bmrb.wisc.edu/metabolomics/)	The central repository for experimental NMR spectral data, primarily for macromolecules	~500 molecules	Structures, structure viewing applets, nomenclature data, extensive 1D and 2D spectral peak lists, raw spectra and FIDs
Fiehn GC-MS Database ( http://fiehnlab.ucdavis.edu/Metabolite-Library-2007/)	The library contains data on compounds for which GC/MS data have been collected	713 compounds	Name, structure, CAS ID*3 of compounds; Spectra and retention indices of GC/MS data
Golm Metabolome Database ( http://gmd.mpimp-golm.mpg.de/)	A public access to custom GC/MS libraries	n/a	Mass spectral and retention time index libraries
HMDB ( http://www.hmdb.ca)	Human Metabolome Database;	More than 6,500;	Chemical data; Clinical data; Molecular Biology/Biochemistry data
	Small molecule metabolites	Link to ~ 1,500 protein and DNA sequences	
Massbank ( http://www.massbank.jp/)	A mass spectral database of experimentally acquired high resolution MS spectral of metabolites	>13,000 spectra from 1,900 different compounds	Very detailed MS data and excellent spectral/structure searching utilities
Metlin ( http://metlin.scripps.edu/index.php)	A repository for mass spectra metabolite data	15,000 structures including more than 8,000 di and tripeptides	MS/MS, LC/MS *^4^ and FTMS*^5^ data
MMCD ( http://mmcd.nmrfam.wisc.edu/)	The Madison Metabolomics Consortium Database (MMCD)	~10,000 metabolite entries; experimental spectral data on ~500 compounds	Chemical formula, names and synonyms, structure, physical and chemical properties; NMR and MS data of compounds, NMR chemical shifts, etc.

Abbreviations: *1NMR: nuclear magnetic resonance; *2-D-HJ: 2 dimensional 1H- jump-and –return pulse spectrum; *3 CAS ID: compound ID assigned by chemical abstract service; *4 LC/MS: liquid chromatograph/mass spectrometry; *5 FTMS: Fourier transform mass spectrometry.

The integration of metabolomics with genomics, transcriptomics, and proteomics plays an important role in the research of systems biology. In many cases mRNA expression and protein levels are poorly correlated, and in certain cases they may be even more poorly correlated with phenotypes which are several steps “up-stream” of the flow of genetic information. Besides, transcriptomics studies can be the quite costly, in contrast, proteomic studies are relatively time consuming. Meanwhile, the application of metabolomics to a mouse model of disease can rapidly profile a range of tissues. This profiling would allow us to understand how one gene mutation affects metabolism in the blood plasma and multiple tissues such as white adipose tissue, fast- and slow- twitch skeletal muscle, and cardiac tissue. As result, an *in vivo* model can be built on systemic metabolism changes across the organism following a genetic modification [14], not all of those findings in transgenic models can be translated into the human diseases yet. In particular, recent progress has been made toward the understanding of the highlighted pathways of changed metabolite profiles in diseases. In this article, we focus to review the applications of metabolomics, and the changes of metabolites as well as the specific pathways underlining the pathophysiology in atherosclerosis, metabolism syndrome, and obesity.

### Applications of metabolomics in atherosclerosis

Metabolomics has being applied to analyze atherosclerotic samples for biomarker discovery and circulating metabolite profiling that may play an important role as regulatory signals in the development of the disease. In order to identify differences in plasma metabolites of individuals with and without ischemia, Sabatine *et al.*[15] employed a MS-based technology in the study. The results showed that lactic acid and its metabolites, such as hypoxanthine, inosine, and alanine that are involve in skeletal muscle AMP catabolism increase after exercise stress test in both patients and healthy controls. In contrast, there was significant discordant regulation of multiple metabolites in patients with ischemia demonstrated by increased MET193 and MET221 and decreased plasma levels of γ-aminobutyric acid, MET288, oxaloacetate, citrulline, and argininosuccinate, which were caused by the preservation of citric acid cycle intermediates to defend ATP production in the myocardium. This metabolomics study exhibited a discovery of novel biomarkers of acute myocardial ischemia. In addition, pattern-recognition techniques were also applied to ^1^H-NMR spectra of human serum. The study showed that the most influential factors for the development of severe atherosclerosis are increased metabolites of CH_2_ chain (chemical shift ð1.26, ð1.3 and ð1.34 on 600 MHz ^1^HNMR spectra) and CH_3_ group (chemical shift ð86) produced from fatty acid side chains in lipid, in particular LDL and VLDL). In this study, investigators can correctly diagnose not only the presence, but also the severity, of angiographically-defined coronary heart disease with a specificity of >90% [16]. The similar serum metabolic profiles and chemical shift patterns were indicated in patients with high systolic blood pressure (SBP), demonstrating that there was a relationship between serum metabolic profiles and blood pressure, which in part was thought due to lipoprotein particle composition differences between the samples [17]. Taken together, these studies showed for the first time a technique capable of providing an accurate, noninvasive, and rapid diagnosis of coronary heart disease and hypertension, which can be used clinically either in population screening or effective targeting of treatments. However, some authors argued about the predictive power and specificity of ^1^H-NMR methodology in the detection of coronary artery disease (CAD), stating that the analysis was dependent mainly on the major lipid regions of the spectra, and that many variables, including gender and drug treatment, affect lipid composition and are potential confounders [18].

Recently, investigators applied GC-MS fingerprinting to the plasma samples of patients with acute coronary syndrome in comparison with that of healthy controls for disease diagnosis with pattern recognition techniques. Interestingly, the results showed that acute coronary syndrome (ACS patients had decreased plasma citric acid, 4-hydroxyproline (4OH-Pro), aspartic acid, and fructose; and increased lactate, urea, glucose, and valine [19]. Though the clear cross-talk of those changes to ACS needs to be further studied, the decrease in plasma 4OH-Pro was especially interesting because circulating 4OH-Pro is thought to prevent the binding of LDL to lipoprotein previously deposited in the vascular wall, as well as releasing already-deposited LDL from therosclerotic lesions. Furthermore, 4OH-Pro is a component of collagen, which also confers stability to the atherosclerotic plaque in the vessels.

In a similar study [20], the investigators applied both GC-MS and ^1^H-NMR to investigate the plasma of patients with stable carotid atherosclerosis in comparison to that of healthy subjects. The results showed that these techniques complement each other and enable a clearer picture of the biological samples to be interpreted not only for classification purposes, but also more importantly to define the metabolic state of patients with carotid atherosclerosis. The metabolomic profiles showed at least 24 metabolites that were significantly modified in the group of atherosclerotic patients by this “nontargeted” procedure. Those metabolites include, but not exclusively, increased _D_-glucose, and decreased _D_-fructose, pyruvate (pyr) and myoinositol (myo) which are involved in impaired insulin sensitivity, glycolysis, and glycogenogenesis; increased 3-OH—butyrate (3HB), acetoacetate (AcAc), long chain-fatty acids (such as palmitate, stearate, 11-transoctadecenoate, and linoleate), _D_-glycerol (gol), and decreased medium-chain fatty acids (hexanoate and laurate) produced from increased lipolysis and decreased lipogenesis, β-oxidation of fatty acids and deficient insulin signaling. In addition, increased 2-hydroxybutyrate was also found in this study, of which the increase was believed due to decreased L-threonine. Since most of the changes are associated to alterations of the metabolism characteristics of insulin resistance, the metabolic profiling in carotid atherosclerosis can be strongly related to metabolic syndrome. In summary, these studies highlight two important points. First, GC-MS and ^1^H NMR fingerprints can provide complementary information in the identification of altered metabolic pathways in patients with carotid atherosclerosis. Second, correlations among results from both techniques, instead of a single study, can provide a deep insight into the patient state.

A number of laboratories have applied metabolomics to understand the progression of atherosclerosis in the apolipoprotein E (ApoE)*3Leiden mouse or the atherogenic ApoE gene knock-out (ApoE^-/-^) mouse model [21,22]. In the ApoE3-Leiden (APO*E3) transgenic mice, which develop only mild type I and II atherosclerotic lesions, there are significant increases in liver-type fatty acid binding protein (L-FABP), in triglycerides, and selected bioactive lysophosphatidylcholine compared to controls, indicating a rapid identification of early protein and metabolite markers of disease pathology of this model. A correlation analysis of identified genes, proteins, and lipids was used to construct an interaction network, though some identified changes needed to be “translated” into human disease. Taken together, these results indicate that integrative biology is a powerful tool for rapid identification of early markers and key components of pathophysiological processes, and constitute the first application of this approach to a mammalian system [21]. Furthermore, the metabolic profiles fulfilled with LC-MS/MS also revealed that 1-Cys peroxiredoxin (1-cyc prx), a novel antioxidant conferring protection against oxidative membrane damage, was predominantly present as reduced protein in apolipoprotein E^+/+^ vessels but is oxidized in apolipoprotein E^-/-^ vessels [22], indicating that increased oxidative stress plays an important role in the development of atherosclerosis in this model. To identify the changes of protein and metabolite in the vessels of ApoE^-/-^ mice on normal chow diet using proteomic and metabolomic technologies [22], investigators analyzed and mapped the protein expression in aortic smooth muscle cell (SMC)s of mice using two-dimensional gel electrophoresis (2-DE), matrix-assisted laser desorption-ionization time-of-flight mass spectrometry (MALDI-TOF MS) and LC-MS/MS [23]. In this study, 79 protein species were identified, of which the expression levels were increased during various stages of atherogenesis. Immunoglobulin deposition, redox imbalance, and impaired energy metabolism were found to precede lesion formation in ApoE^-/-^ mice. Meanwhile, NMR revealed a decline in alanine and a depletion of the adenosine nucleotide pool in vessels of 10-week-old ApoE^-/-^ mice. Furthermore, attenuation of lesion formation was associated with alterations of NADPH generating malic enzyme, which provides reducing equivalents for lipid synthesis and glutathione recycling, and successful replenishment of the vascular energy pool [23]. Given that increased dietary cholesterol is associated with atherogenesis in man and mouse, in a combined transcriptomics and metabolomic study, investigators [24] used novel whole-genome and HPLC/MS-based metabolome technologies to investigate the proinflammatory components of atherosclerosis that originate in livers of the groups of ApoE*3Leiden mice fed with cholesterol-free (control), low cholesterol (LC; 0.25%), and high cholesterol (HC; 1%) diets. The results showed that induced atherosclerosis was proportional to dietary intake of cholesterol because HC stress significantly activated specific proinflammatory pathways that is the platelet-derived growth factor (PDGF), interferon-γ (IFNγ), interleukin-1 (IL-1) and tumor necrosis factor-α (TNF-α) signaling pathways. Activation of these proinflammatory pathways with HC treatment leads to a significant up-regulation of regulators, such as mitogen-activated protein kinases (MAP kinases), complement factors, and acute phase proteins such as serum amyloid A (SAA). Notably, several of these regulators control both lipid metabolism and inflammation, which suggests that the high-cholesterol diet not only induces inflammation but also alters lipid metabolism, thereby linking the dyslipidemia and inflammation with the early lesion formation of atherogenesis with this animal model Apparently, the study demonstrated that the combination of transcriptomics and metabolomics is a newly developed, functional systems biology tool, which allows for better definition of the perturbations associated with a given dietary intervention and to identify the novel regulatory pathways and transcriptional regulators controlling both lipid metabolism and inflammatory responses.

The usefulness of metabolomics in pathology investigation was further cemented, in which NMR-based metabolomics of mouse urine was used in conjunction with the traditional staining and imaging of aortas for the characterization of disease advancement, that is, plaque formation in untreated and angiotensin I-converting enzyme inhibitor Captopril-treated apoE^-/-^ mice [25]. The result showed that xanthine and ascorbate were elevated in the untreated mice. The increased xanthine level in the untreated group was thought as a marker for the plaque formation in apoE^-/-^ mice. Meanwhile, the elevated ascorbate in the untreated group is consistent with findings that stress will cause more ascorbate to be produced, be it to counter greater ROS or for vasodilation. Interestingly, the metabolomics approach with multivariate analysis was able to differentiate the Captopril-treated from the untreated mice in general agreement with the staining results. Metabolites were identified to be indicative of disease progression because of their role in oxidative stress. Plasma and urine samples from atherosclerotic and control rats have been compared by ultra-fast liquid chromatography coupled to ion trap-time of flight (IT-TOF) mass spectrometry (UFLC/MS-IT-TOF) and 12 metabolites in rat plasma and 8 metabolites in urine were identified as potential biomarkers. Concentrations of leucine, phenylalanine, tryptophan, acetylcarnitine, butyrylcarnitine, propionylcarnitine, and spermine in plasma and 3-O-methyl-dopa, ethyl N2-acetyl-L-argininate, leucylproline, glucuronate, t6A N^6^-(N-threonylcarbonyl)-adenosine and methyl-hippuric acid in urine decreased in atherosclerotic rats. Meanwhile, ursodeoxycholic acid, chenodeoxycholic acid, lyso-phosphatidylcholines (lysoPC) C16:0, lysoPC C18:0 and lysoPC C18:1 in plasma and hippuric acid in urine were in higher levels in atherosclerotic rats. The altered metabolites demonstrated abnormal metabolism of phenylalanine, tryptophan, bile acids and amino acids. This report indicates that metabolomics is a promising tool for disease research [26].

### Applications of metabolomics in diabetes and obesity

The development of T2DM and obesity are highly correlated with uptake of the high fat diet. These disorders result in a number of metabolic perturbations including imbalance of glucose metabolism and dyslipidemia in key organs and blood plasma. These alterations lead to changes in the proportions of very low density lipoprotein (VLDL)/low density lipoprotein (LDL) to high density lipoprotein (HDL) and induce atherosclerosis. The disorders interact to produce what has been referred to as a Metabolic Syndrome whereby a number of separate risk factors for atherosclerosis interact to greatly magnify the risk of developing cardiovascular disease. Thus, metabolic changes are central to these disorders, and we would expect that any process aimed at measuring global metabolism changes would provide a good description of the phenotype of these diseases. Therefore, metabolomics has been used to identify the potential new disease markers of diabetes and obesity. In one study, LC-MS followed by multivariate statistical analysis was successfully applied to the metabolic profiling of plasma phospholipids in T2DM. The specific phospholipid molecular species, such as glycerophosphophoethanolamine (GPE), diacyl phophatidylethanolamine, and lyso-phosphatidylcholines (Lyso-PC C16:0 and C18:0), detected at m/z range from 480 to 788 on LC-MS were identified as potential biomarkers for classifying the DM2 patients from the controls. With this methodology, it was possible not only to differentiate the T2DM from the controls but also to identify the potential biomarkers with LC-MS/MS. The proposed method shows that LC-MS combined with multivariate statistical analysis is a complement or an alternative to NMR for metabolomics applications [7]. In the Insulin Resistance Atherosclerosis Study (IRAS), in which lipoprotein particles were measured using NMR, the investigator showed that a range of lipoprotein abnormalities in prediabetic individuals, including compositional changes in HDL (decreased) and VLDL (increased). These findings extend previous work indicating a proatherogenic state in healthy, nondiabetic subjects who subsequently develop diabetes [8], revealing that declines in glycerol and leucine/isoleucine (markers of lipolysis and proteolysis, respectively) jointly provide a predictor of insulin sensitivity [27,28]. In a clinical study with type 1 diabetic (T1D) humans, MS revealed significant perturbations in the levels of plasma amino acids and amino acid metabolites during insulin deprivation as well as several metabolic pathways like protein synthesis and breakdown, gluconeogenesis, ketogenesis, amino acid oxidation, mitochondrial bioenergetics, and oxidative stress are also perturbed [29]. An ultra-performance LC quadruple–time of flight mass spectrometry (UPLC-q-TOF-MS)-driven non-targeted metabonomics study showed considerable changes of metabolite fingerprints in pre-diabetes, including increases in fatty acid- (FFAs C18:2, C16:1, C20:4 and C22:4; saturated FFA palmitate and FFA stearate, unsaturated FFA Oleate) and in glycochenodeoxycholic acid; and decrease in Lyso-PC- metabolism, uric acid, as well as tricarboxylic acid cycle -metabolism (TCA cycle) [30]. In another clinical study using targeted LC-MS profiling of blood plasma, investigators aimed to investigate whether metabolite profiles could predict the development of diabetes. The results showed a highly significant association between the levels of five branched chain and aromatic amino acids isoleucine, leucine, valine, tyrosine, and phenylalanine and the probability of future onset of diabetes. A combination of three amino acids predicted future diabetes (with a more than fivefold higher risk for individuals in top quartile). The results were later replicated in an independent, prospective cohort. These findings underscore the key role of amino acid metabolism early in the pathogenesis of diabetes and suggest that amino acid profiles could aid in diabetes risk assessment [31]. Furthermore, examination of plasma esterified fatty acids (EFAs) and non-esterified fatty acids (NEFAs) metabolic profiling using GC-MS indicated that most of the arachidonic acids (the class of C20 FAs) had significantly distinguishing characteristics in pathological progression. Most likely, because arachidonic acids are involved in in the anabolism of prostaglandins, these molecules are important modulators of inflammatory processes. Besides, most of the plasma EFA concentrations were reduced in DM cases when compared with control group, while NEFAs increased significantly. The EFA level fluctuation was suspected to be related with the cellular self-repair mechanism and NEFA variation resulting from EFAs self-adjusting mechanism. These findings could indicate potential biomarkers for monitoring the progression of DM as well as diabetic nephropathy (DN) [32].

This targeted approach using gas-chromatography-mass spectrometry has also been applied to insulin resistance studies *in vivo* and an increase in phenylalanine metabolites was in agreement with the known regulation of the phenylalanine hydroxylase gene by Hnf1α [33]. MS-based metabolic profiling of acylcarnitines species identified an increase in the concentration of lipid-derived β-hydroxybutyrate in muscle of mice that overexpress hepatic malonyl-CoA decarboxylase and exhibit improved insulin resistance [10]. Meanwhile, in wild-type mice fed with a high-fat diet, significant changes to metabolites in the liver were identified [34], including increases in urea cycle intermediates, consistent with increased deamination of amino acids used for gluconeogenesis, and 1,5-anhydroglucitol, a previously identified marker of short-term glycemic control. Of the identifiable metabolites in plasma, wild-type mice fed with the high-fat diet also had increases in plasma stearate and two pyrimidine-related metabolites. These changes are consistent with increased deamination of amino acids used for gluconeogenesis [34]. The findings are also supported by a study in T2D rats and streptozotocin-induced diabetic rats (a type I diabetes model) [35,36]. The metabolic effect of the PPARγ activation and PPARδ activation in regulating metabolism in adipose tissue and insulin sensitivity was well-defined *in vivo* in white adipose tissue from ob/ob mice (mice homozygous for the obese spontaneous mutation of leptin gene) and *in vitro* in cultured 3T3-L1 adipocytes using ^1^H NMR and MS metabolomics. The metabolic effects of the receptors were readily distinguished, with PPARγ activation characterized by increased fat storage, synthesis and elongation, while PPARδ activation caused increased fatty acid β-oxidation, tricarboxylic acid cycle rate and oxidation of extracellular branch chain amino acids. Stimulated glycolysis and increased fatty acid desaturation were common pathways for the agonists. PPARγ and PPARδ restore insulin sensitivity through varying mechanisms. PPARδ activation increases total oxidative metabolism in white adipose tissue, a tissue not traditionally thought of as oxidative. However, the increased metabolism of branch chain amino acids may provide a mechanism for muscle atrophy, which has been linked to activation of this nuclear receptor. PPARδ has a role as an anti-obesity target and as an anti-diabetic target, and hence may target both the cause and consequences of dyslipidemia [9]. These results were supported by a recent review that defined the role PPARδ in regulating fatty-acid oxidation in adipose tissue and the interaction between aging and PPARα in liver [37]. Obese individuals with or without T2D are characterized by dysregulated fatty acid and amino acid metabolism. Recent investigations have applied comprehensive metabolomics profiling to gain a broad understanding of the metabolic differences between lean, obese, and diabetic individuals. An investigator demonstrated, using targeted metabolomics, elevations in acylcarnitines (AcyCN) and amino acid concentrations; in particular branched chain amino acids and C3, C5, C6, and C8:1 acylcarnitines [12]. Furthermore, overweight/obese men showed a higher proportion of stearic acid and lower proportion of oleic acid in serum phospholipids. Additionally, overweight/obese individuals had higher fat intake and lower ratios of polyunsaturated fatty acids to saturated fatty acids when compared to healthy controls. Three lysoPCs were identified as potential plasma markers and the study confirmed eight known metabolites for overweight/obesity men as overweight/obese subjects showed higher levels of lysoPC C14:0 and lysoPC C18:0 and lower levels of lysoPC C18:1 than lean subjects. The result indicates an abnormal metabolism of two branched-chain amino acids (BCAA), two aromatic amino acids, and fatty acid synthesis and oxidation [38,39]. Recent studies have associated the compromised insulin signaling in patients with obesity, prediabetes, and T2DM with altered intermediary metabolism of fats and amino acids. The increase in blood concentrations of selected essential amino acids and their derivatives, in particular, BCAA, and/or carnitine esters derived from partial BCAA catabolism, sulfur amino acids, tyrosine, and phenylalanine, are apparent with obesity and insulin resistance, often before the onset of clinically diagnosed T2DM [40]. The decreases in the metabolism of essential fatty acids and polyunsaturated fatty acids including eicosapentaenoic acid (EPA), docosahexaenoic acid (DHA), and arachidonic acid (AA) are believed to play a significant role in the pathophysiology of metabolic syndrome and diabetes mellitus [41]. A targeted metabolomic investigation of the plasma pattern of families burdened with early onset cardiovascular disease revealed decreased linoleic acid (LA) and AA as biomarkers [42] because the adequate amounts of EPA, DHA, LA, and AA synthesized and released by ECs can prevent aggregation of platelets on their surface and decrease the expression of adhesion molecules and production of pro-inflammatory cytokines, such as IL-1β, IL-2,IL-6 and TNF-α, so that atherosclerosis would not occur. Of note, bile acids (BAs) are now discussed as metabolic integrators of whole-body energy homeostasis. In fact, they were among the metabolites showing the most striking change during oral glucose tolerance test (OGTT) in a targeted metabolic profiling approach [27] and in a non-targeted metabolomics study [43] though their characteristic biphasic alterations (increase-decrease-increase pattern) during OGTT needs to be better understood. Also pertinent, BAs can influence glucose and lipid metabolism [44] through the activation of farnesoid X receptor (FXR), a member of the superfamily of ligand-activated nuclear receptor transcription factors, lowers plasma triglyceride synthesis by a mechanism that may involve the repression of hepatic sterol regulatory element-binding protein (SREBP)-1c expression and/or the modulation of glucose-induced lipogenic genes. FXR^-/-^ mice display both impaired glucose tolerance and decreased insulin sensitivity, therefore, the findings suggest that FXR activity can be a potential biomarker of the development and treatment of metabolic syndrome and T2D.

### Potential mechanisms and the signaling pathways underlying the metabolite profiling changes associated with atherosclerosis, diabetes, and obesity

The mechanisms associated with accelerated atherosclerosis development seen with the insulin resistance-associated conditions like T2DM and obesity are still under investigation, but it is believed that a decline in the bioavailability of nitric oxide (NO) and an increase in reactive oxygen species (ROS) are the most crucial factors. NO has the functions of vasorelaxant [45], anti-inflammatory [46], antiproliferative [47], antioxidant [48], and antiplatelet actions [49], demonstrating a crucial role in preventing endothelial dysfunction. The decline in the bioavailability of NO occurs as a result of reduced biosynthesis of NO and/or increased degradation by ROS. Several pieces of evidence have shown that impaired NO-dependent vasodilatation can predict future cardiac events and the development of coronary artery disease [50,51]. A range of studies have provided compelling evidence supporting a strong association between obesity, insulin resistance, and NO bioavailability [52-59]. Furthermore, in a diet-induced model of obesity the data has shown that insulin-mediated NO release is blunted in the early development of obesity [60], which is believed to be associated with increased endothelial cell-derived ROS. This finding was supported by a study in a non-obese model of whole body insulin resistance, where reduced basal and insulin-mediated NO release was demonstrated [61], as a result of increased endothelial cell nicotinamide adenine dinucleotide phosphate (NADPH) oxidase-derived ROS from vascular oxidative stress [62]. Oxidative stress is thought to play a pivotal role in the pathophysiology of atherosclerosis [63], and insulin resistance [64]. A prevailing theory is that increased delivery of lipids to muscle tissues saturates the capacity for mitochondrial ß-oxidation, leading to accumulation of bioactive lipid-derived metabolites such as diacylglycerols and ceramides in the extra mitochondrial space and the activation of stress/serine kinases that interfere with insulin action. More recent studies have shown that fatty acid oxidation is actually increased in muscle tissues in response to high-fat feeding, but with no corresponding increase in TCA cycle activity. This results in accumulation of incompletely oxidized lipids in the mitochondria and depletion of TCA cycle intermediates, possibly resulting in mitochondrial stress and interference with insulin actions [65].

ROS are thought to promote atherosclerosis through a number of different mechanisms including enhanced oxidation of lipoproteins, activation of pro-inflammatory genes, alteration of vascular smooth muscle cell phenotype, and possibly most importantly, reduction of NO bioavailability. ROS in the arterial wall are generated by several enzymes including NADPH oxidase [66], xanthine dehydrogenase; cytochrome p450 based enzymes, the mitochondrial electron transfer chain, as well as infiltrating inflammatory cells [66]. Moreover, NADPH oxidase-derived ROS can impact the major cellular sources of ROS, the mitochondria, to enhance superoxide production from this organelle [67]. As a result of this complex interplay between sources of ROS, a self-propagating cycle can ensue, amplifying endothelial ROS production, reducing NO bioavailability and generating an increasingly proatherosclerotic environment. As discussed above, mitochondrial electron transport is one of the major producers of ROS. It was believed that uncoupling protein 1 (UCP-1) in vascular smooth muscle cells plays an important role in ROS production and atherosclerosis. The study in Semenkovich’s laboratory demonstrated that UCP-1 generated ROS from vascular smooth muscle cells leads to elevated ROS, reduced NO bioavailability and accelerated atherosclerosis in mice on an ApoE deficient background [68]. In contrast, overexpression of UCP-1 in the endothelium was shown to blunt free fatty acid (FFAs)-induced ROS production, suggesting that overexpression of UCP-1 in the endothelium may protect against endothelium cell dysfunction. The controversy warrants future studies. In addition to vascular smooth muscle cells, the role of mitochondrial dysfunction and ROS production in the endothelium during diabetic and obesity states has been extensively examined. It is believed that free fatty acids at pathophysiological concentrations increases ROS production in macrovascular endothelial cells through increased expression of NADPH oxidase-p47 (phox) and endothelial oxidative stress, with selective compensatory upregulation of antioxidant enzymes and Ser1177-phosphorylated endothelial nitric oxide synthase (eNOS) [69]. Consistent with findings in humans, NADPH oxidase has been shown to be a key sources of ROS in adipose tissue of obese mice [70]. Of note, endothelial cell NADPH oxidase is thought to be the principal source of reduced NO bioavailability and significant vascular oxidative stress in insulin resistance [62,71].

Recently, NADPH oxidase has emerged as a major source of superoxide in obese humans [72], those with metabolic syndrome [73], patients with T2D [74], and those with chronic heart failure [75]. Endothelin-1 and nuclear factor-kappaB protein expression also appear to be elevated in obese adults, suggesting a novel insight into the molecular mechanisms linking obesity to increased risk of clinical atherosclerotic diseases in humans [69].

It is well established that obesity and T2DM are associated with proatherogenic dyslipidemia, which is characterized as an increase in plasma triglycerides and FFAs, a reduction in HDL, and the presence of small, susceptible LDL particles. A significant event in the early progression of atherosclerosis is the permeation of small LDL particles across the endothelial barrier and its subsequent accumulation in the vascular wall [76]. The discrete steps in triglyceride and FFAs handling increases ROS production and activates NADPH oxidase and mitochondrial electron chain to generate superoxide. The excessive ROS production from one source can lead to enhanced production of ROS from another. Phospholipids contained within LDLs are highly susceptible to oxidation by ROS leading to the generation of oxidized phospholipids (OxPLs). OxPLs formation triggers endothelial cell activation, facilitating monocyte adhesion and stimulating expression of inflammatory cytokines [77]. Aside from this, elevated FFA itself may also have direct effects on NO biosynthesis [78-80] and impair mitochondrial uncoupling [81]. In insulin-resistant murine models, FFA-induced ROS production substantially inhibited prostacyclin synthase and eNOS activity in aorta [69]. The interlocking effects of FFA on insulin resistance and ROS may lead to a vicious cycle of lipotoxicity-induced vascular dysfunction. Activation of the transcription factor NF-kB signaling pathway and inflammatory mediator toll-like receptor-4 (TLR4) signaling via the serine kinase inhibitor kappaB kinase B (IKKB) activation may disrupt insulin signaling via inhibition of insulin receptor substrate 1, Akt (protein kinase B), and eNOS phosphorylation. In turn, this leads to a decrease in NO production, perhaps instigating inflammation and endothelial dysfunction [82-84].

## Conclusions

**Figure 2:**
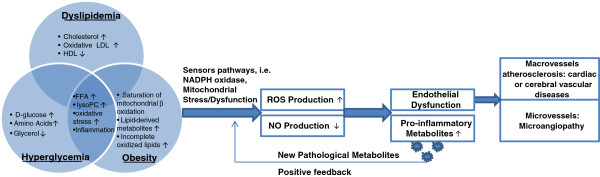
**Metabolome analysis related to hyperglycemia/insulin resistance, obesity, and hyperlipidemia which contribute to the development of endothelial dysfunction.** The reciprocal effects of hyperglycemia/insulin resistance, obesity, and dyslipidemia result in mitochondria stress/dysfunction via a complex of factors including oxidative stress, inflammation, incomplete fat oxidation, etc. As a result, mitochondria dysfunction increases the production of reactive oxygen species (ROS) and decreases nitric oxide (NO), which ultimately produces endothelial dysfunction and progressively causes atherosclerosis in macrovessels and microangiopathy in microvessels.

In summary, as a research tool, metabolomics has been successfully applied to the research of disorders such as atherosclerosis, diabetes, and obesity. This technology has been applied to the identification of novel biomarkers not only for the diagnosis of diseases, but also for the assessment of severity and provision of prognostic information in these conditions. An emerging set of metabolomics tools like MS, NMR, and other technologies enable the monitoring of metabolite profiles from biological samples of these disorders that has provided certain advantages relative to other “omics” technologies like genomics, transcriptomics, and proteomics. Since it measures chemical phenotypes that are the net result of genomic, transcriptomics, and proteomic variability, metabolomics provides the most integrated profile of biological status. However, metabolomics is still a field in its infancy, with some limitations and difficulties and even confusions in the interpretation of its data. In contrast, Genome-wide association studies and mRNA profiling by microarray analysis are relatively mature technologies. Though metabolomics technologies are still under development, they complement other functional “omics” approaches, such as high throughput genome sequencing, RNA expression analysis, and proteomics. Metabolomics promises to be invaluable in illuminating systems biology, the discovery of biomarkers for disease diagnosis, and treatment assessment. Metabolomic studies to date have identified quite diversified metabolites in blood, tissues, and urine in the process of atherosclerosis, diabetes, and obesity, of which most are the intermediates of specific lipid classes, fatty acids, carbohydrates, amino acids, bile acids, purine, pyrimidine, and proteins. The identified metabolites and their sources and/or related mechanisms with atherosclerosis, diabetes and obesity are summarized in Tables 6 and 7. Accumulations of the metabolomic datasets in these studies led us to the mechanism that decreased NO bioavailability, increased ROS production and mitochondria dysfunctions play crucial roles in the production of metabolites associated with these disorders (Figure 2). These findings may narrow the focus of future metabolomic studies in understanding the mechanisms of atherosclerosis, diabetes, and obesity.

**Table 6:** Summary of identified metabolites in atherosclerosis

**Metabolites**	**Change**	**Sources or pathways**	**Species of study**	**PMID**
MET193 , MET221 Metabolites of CH2 chain and CH3 chain fatty acids	↑	CH2 chain and CH3 chain groups of fatty acid in LDL and VLDL	human plasma and human serum	16344383, 12447357, 12572799
glucose, lactate, urea and valine	↑	Carbohydrate and amino acid metabolism	human plasma	20425260, 19813770
3-OH-butyrate, acetoacetate, palmitate, stearate, 11-transoctadecenoate, linoleate, and D-glycerol	↑	Increased lipolysis and decreased lipogenesis, β-oxidation and deficient insulin signaling	human plasma	19813770
2-hydroxybutyrate	↑	Decreased L-threonine	human plasma	19813770
Oxidized 1-Cys- peroxiredoxin	↑	Increased oxidative stress	ApoE -/- mouse and mouse aortic sooth muscle cells	16123314
γ-aminobutyric acid, MET288, citrulline, argininosuccinate, oxaloacetate	↓	Preservation of citric acid metabolism intermediates	human-plasma	16344383
4-hydroxyproline (4OH-Pro), citric acid, aspartic acid, fructose,	↓	Amino acid and carbohydrate metabolism, deficient protection of 4OH-pro in vascular wall	human plasma	19813770 20425260
D-fructose, pyruvate, myoinostol , medium-chain fatty acids (hexanoate and laurate)	↓	Impaired insulin sensitivity, glycolysis and glycogenogenesis; increased lipolysis and decreased lipogenesis	human plasma	19813770
reduced 1-Cys peroxiredoxin, alanine, and NADPH generating malic enzyme	↓	Increased oxidative stress Reduced equivalents for lipid synthesis and glutathione recycling	ApoE -/- mouse and mouse aortic smooth muscle cells	16123314 16240290
Cholesterol, LDL and VLDL	↑	Activated inflammatory pathways, then upregulation of regulators: mitogen-activated protein kinases	ApoE*3Leiden mouse ApoE -/- mouse	16123314 17892536
Xanthine, ascorbate	↑	Plaque formation of vessels Stress induced more ascorbate production and oxidative stress	ApoE -/- mouse urine	19565469
Ursodeoxycholic acid, chenodeoxycholic acid, lysoPC (C16:0, C18:0, C18:1)	↑	Abnormal metabolism of bile acids and amino acids	atherosclerosis rat plasma	19576453
Hippuric acid	↑	Abnormal metabolism of bile acids and amino acids	atherosclerosis rat urine	19576453
HDL	↓	Decreased inhibition of inflammatory pathways	ApoE*3Leiden mouse ApoE -/- mouse	16123314 17892536
Leucine, phenylalanine, tryptophan, acetylcarnitine, butyrylcarnitine, propionylcarnitine, spermine	↓	Abnormal metabolism of amino acids	Atherosclerotic rat plasma	19576453
3-O-Methyl-dopa, ethyl N2-acetyl-L-argininate, leucylproline, glucuronate, N6-threonylcarbonyl-adenosin and methyl-hippuric acid	↓	Abnormal metabolism of amino acids, phenylalanine, tryptophan and bile acids	atherosclerosis rat urine	19576453

↑: Increased; ↓: Decreased.

**Table 7:** Summary of identified metabolites in diabetes or obesity

**Metabolites**	**Change**	**Sources or pathway**	**Species of study**	**PMID**
Glyceriphophphoetanolami-ne, diacyl-phophatidylethanolamine	↑	Impaired phospholipid metabolism	diabetic patient plasma	15987116
Lyso-PCs^*^	↑	Lipid metabolism	diabetic patient plasma	15987116
VLDL^*^	↑	Lipoprotein abnormalities	Pre-diabetic patient plasma	15983261
Fatty acids (C18:2, C16:1, C20:4 and C22:4); saturated free fatty acid palmitate and stearate,; unsaturated Oleate-.	↑	Decreased metabolism of LysoPC^*^, uric acid and tricarboxylic acid cycle	Pre-diabetic patient plasma	20676218
Arachidonic acids, non-esterified fatty acids	↑	Anabolism of prostaglandins Modulators of inflammatory processes	diabetic patient plasma	21338761
isoleucine, leucine, valine, tyrosine, and phenylalanine	↑	Abnormal metabolism of branched chain and aromatic amino acid	Pre-diabetic and diabetic patient plasma	21423183
HDL^*^	↓	Lipoprotein abnormalities	Pre-diabetic patient plasma	15983261
Glycerol, leucine/isoleucine	↓	Lipolysis and proteolysis,	Pre-diabetic patient plasma	18682704, 20976215
Esterified fatty acids	↓	Cellular self-repair mechanism	diabetic patient plasma	21338761
phenylalanine	↑	Impaired regulation of the phenylalanine hydroxylase gene by Hnf1α gene	Insulin resistance patient, Type 2 diabetes mellitus mouse (Hnf1α-null mouse) and rats, type 1 diabetic rats	20943816, 20150186, 21440515, 22546713
C3, C5, C6, and C8:1 acylcarnitines	↑	Increased catabolism of a branched-chain amino acid	Obesity patients serum	19356713
Stearic acid	↑	Change of serum phospholipid contents	Obesity patients serum	20560578
LysoPC^*^ (C14:0, C18:0)	↑	Abnormal metabolism of BCAA^*^, aromatic amino acids, and fatty acid synthesis and oxidation	Overweight/obesity human	20560578 22266733
Sulfur amino acids, tyrosine	↑	Partial BCAA catabolism	Metabolic syndrome patient	19357637
Farnesoid X receptor	↑	Superfamily of ligand-activated nuclear receptor transcription factors	Impaired glucose tolerance and insulin resistance patient	19126757
Oleic acid	↓	Change of serum phospholipid contents	Obesity patients serum	20560578
LysoPC^*^ (C18:1)	↓	Abnormal metabolism of BCAA^*^, aromatic amino acids, and fatty acid synthesis and oxidation	Overweight/obesity human	20560578 22266733
Eicosapentaenoic acid, docosahexaenoic acid, arachidonic acid	↓	Metabolism of essential fatty acids and polyunsaturated fatty acids	Patient of metabolic syndrome and diabetes mellitus	16892270

↑: Increased; ↓: Decreased.

## Methods

All procedures of animal studies in cited papers were performed according to the protocols approved by the institutional committee for use and care of Lab animals [21,22]. The protocols of human studies were approved by the ethical committee of the department of medicine or human research committee [45,58].

## Consent

Written informed consent was obtained from the patient for publication of this report and any accompanying images.

## Competing interests

The author(s) declare that they have no competing interests.

## Authors’ contributions

FYD: has drafted the manuscript. AV: has participated in drafting the manuscript. HW: has participated in drafting and revising the manuscript. XFY: has participated in drafting and revising the manuscript, and gave the final approval of the version to be published. All authors read and approved the final manuscript.
